# Attention-Dependent Modulation of Cortical Taste Circuits Revealed by Granger Causality with Signal-Dependent Noise

**DOI:** 10.1371/journal.pcbi.1003265

**Published:** 2013-10-24

**Authors:** Qiang Luo, Tian Ge, Fabian Grabenhorst, Jianfeng Feng, Edmund T. Rolls

**Affiliations:** 1 Shanghai Center for Mathematical Sciences, Fudan University, Shanghai, PR China; 2 College of Information Systems and Management, National University of Defense Technology, Hunan, PR China; 3 Centre for Computational Systems Biology, School of Mathematical Sciences, Fudan University, Shanghai, PR China; 4 Department of Computer Science, University of Warwick, Coventry, United Kingdom; 5 Department of Physiology, Development and Neuroscience, University of Cambridge, Cambridge, United Kingdom; 6 Oxford Centre for Computational Neuroscience, Oxford, United Kingdom; Indiana University, United States of America

## Abstract

We show, for the first time, that in cortical areas, for example the insular, orbitofrontal, and lateral prefrontal cortex, there is signal-dependent noise in the fMRI blood-oxygen level dependent (BOLD) time series, with the variance of the noise increasing approximately linearly with the square of the signal. Classical Granger causal models are based on autoregressive models with time invariant covariance structure, and thus do not take this signal-dependent noise into account. To address this limitation, here we describe a Granger causal model with signal-dependent noise, and a novel, likelihood ratio test for causal inferences. We apply this approach to the data from an fMRI study to investigate the source of the top-down attentional control of taste intensity and taste pleasantness processing. The Granger causality with signal-dependent noise analysis reveals effects not identified by classical Granger causal analysis. In particular, there is a top-down effect from the posterior lateral prefrontal cortex to the insular taste cortex during attention to intensity but not to pleasantness, and there is a top-down effect from the anterior and posterior lateral prefrontal cortex to the orbitofrontal cortex during attention to pleasantness but not to intensity. In addition, there is stronger forward effective connectivity from the insular taste cortex to the orbitofrontal cortex during attention to pleasantness than during attention to intensity. These findings indicate the importance of explicitly modeling signal-dependent noise in functional neuroimaging, and reveal some of the processes involved in a biased activation theory of selective attention.

## Author Summary

We show that in cortical areas such as the insular, orbitofrontal, and lateral prefrontal cortex, the variation of the blood-oxygen level dependent (BOLD) time series across trials measured with functional magnetic resonance imaging (fMRI) increases with the magnitude of the signal. We describe a new method of measuring causal effects with Granger causality that takes into account this signal-dependent noise. We show in a functional neuroimaging investigation with the new method that there is a causal influence from the anterior lateral prefrontal cortex that during attention to the pleasantness of taste stimuli increases the response of the orbitofrontal cortex to the taste; and there is a causal influence from the posterior lateral prefrontal cortex to the insular taste cortex during attention to the intensity of taste stimuli. This shows how part of the circuitry involved in the effects of selective attention on the pleasantness and intensity of stimuli operates in the brain.

## Introduction

In the past decade, Granger causality (GC) has emerged as a widely used method for causal inferences, and has been applied to biological time series obtained from many different types of investigation, for example, to the fMRI blood-oxygen level dependent (BOLD) signals to detect effective connectivity between brain areas and thus to shed light on how the brain works [1]–[4]. The basic idea of GC can be traced back to Wiener [5], who conceived the notion that if the prediction of one time series can be improved by incorporating the past history of a second one, then the second time series has a causal influence on the first. Granger later formulated this idea in the context of linear autoregressive (AR) models [6]. GC is completely data-driven and based on time precedence. The interactions discovered by GC may be unidirectional or reciprocal. GC is easy to implement, relies on a small set of straightforward assumptions, and does not need any knowledge about how the data are generated. Therefore, it can be applied directly to almost any time series data [7]. However, over-simplification of the model may result in an incorrect use or interpretation of GC and even spurious causal inferences in some situations [8]–[10]. Care is therefore needed in the use of GC.

One possible over-simplification in some scenarios is that the covariance matrix of the noise, conditional on the past history of the time series and the noise process, is assumed to be time invariant. For example, spike trains of neurons are typically close to Poisson processes in their timing, and the variance thus increases linearly with the signal [11], [12]. Similar conditionally heteroskedastic data have been observed in many physiological recordings, such as the data collected from patients with epilepsy and Parkinson's disease [13]. Therefore, it is natural to conjecture that changes in the volatility of one time series may have an impact on the mean activity or volatility of another time series, which indicates that causal influences may be evident in the second order statistics. Clearly, these causal relationships cannot be captured by classical GC based on a simple AR model, which does not deal with time series data with changing volatility (variance). Moreover, although it has been widely observed and investigated that the signal-dependent noise plays important roles in neuronal activities [14]–[16], it is still unclear whether this property carries through to fMRI BOLD signals, after the neuronal signals are delayed and smoothed by the haemodynamic response function.

In this paper, we provide empirical evidence that the variance of the noise in the fMRI BOLD time series increases linearly with the square of the signal in a number of cortical areas, such as the insular taste, orbitofrontal, and lateral prefrontal cortical areas. In this context we present a Granger causal model with signal-dependent noise to detect GC in both the mean and variance of data with time varying volatility. We also propose a likelihood ratio test to infer GC with signal-dependent noise accurately and efficiently. We show, by simulation studies, that this novel method substantially outperforms classical GC when signal-dependent noise is present.

The new method is evaluated with an fMRI investigation [17] to identify the source of the top-down selective attentional control that differentially biases brain systems involved in affective vs sensory analysis [17]–[19]. Instructions to pay attention to and later rate the pleasantness of a taste increased the activations to taste stimuli measured with fMRI in the orbitofrontal and pregenual cingulate cortices [17], where the subjective pleasantness of taste is represented [20]–[24], but not the primary taste cortex in the anterior insula [17], where the subjective intensity and identity of taste are represented [20]–[22], [24]–[26]. Instructions to pay attention to and later rate the intensity of a taste increased the activations to taste in the insular taste cortex but not in the orbitofrontal and pregenual cingulate cortices [17]. Our new method reveals how the effective top-down connectivity changes when attention is paid to the pleasantness vs the intensity of a taste, and helps in the interpretation of the source of the signals that implement top-down attention.

## Materials and Methods

### Granger causality with signal-dependent noise

#### Classical Granger causality

We start with a brief review of classical GC. Consider the following zero-mean vector autoregressive model (VAR) of order 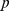:
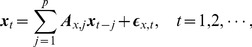
(1)where 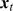 is a 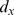-dimensional column random vector, 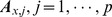 are fixed 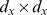 coefficient matrices, and 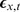 is a 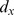-dimensional independent identically distributed (i.i.d) white noise or innovation process, with a positive definite and time invariant covariance matrix 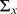. We require that this VAR(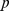) process is stable, that is,
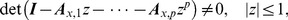
(2)where 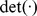 is the determinant of a matrix, 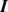 is an identity matrix, and 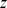 is a complex variable. This stability condition implies that the VAR(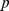) process is weakly stationary, i.e., its first and second order moments exist and are time invariant [27].

Now, assume 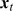 and 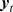 admit a jointly stable VAR representation. 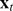 can thus be modeled as
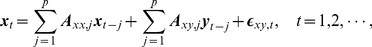
(3)where 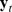 is a 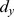-dimensional column random vector, and 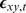 is a white noise process with a covariance matrix 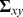.

Classical GC depends on temporal precedence and predictability. The idea is that a cause cannot come after the effect. Thus, if 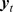 affects 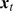, including the past information of 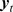 should improve the predictions of 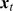. More formally, if the prediction error of 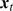 is reduced when the past information of 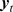 is taken into account, then 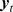 has a causal influence on 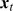 in the sense of Granger. Formulating the idea in the context of a VAR model, the causal influence from 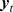 to 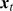 in the time domain can be quantified as [28], [29]

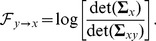
(4)

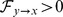
 indicates a causal influence from 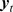 to 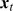, and 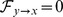 otherwise. Note that model (1) is a restricted version of model (3), and that 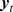 does not cause 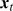 if and only if 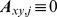 for all 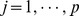 [27].

When the white noise is Gaussian distributed, it has been shown that the GC measure in Eq. (4) is equivalent to the likelihood ratio test statistic [30]

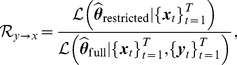
(5)where 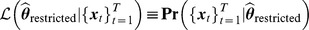 is the likelihood function, i.e., the probability of the observed time series 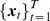, given the maximum likelihood estimate of the parameters 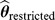 of the restricted model (1). 
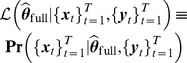
 is interpreted similarly under the full model (3). Therefore, a likelihood ratio test can be used for causal inference:
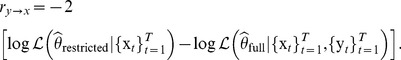
(6)The test statistic 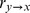 is approximately chi-squared distributed, with degrees of freedom 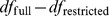, where 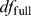 and 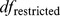 are the number of free parameters of the full model (3) and the restricted model (1), respectively.

#### The signal-dependent noise model

To relax the assumption of a time invariant covariance matrix in the AR model, Engle invented the first changing volatility model — the autoregressive conditional heteroskedasticity (ARCH) model [31], which was then extended to generalized ARCH (GARCH) models [32], [33] as well as multivariate cases [34]–[36]. Assume 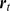 is a 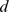-dimensional zero mean, serially uncorrelated process, which may be the residual process of some dynamic model and can be represented as
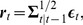
(7)where 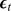 is a 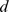-dimensional i.i.d white noise process, 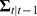 is the conditional covariance matrix of 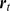, given 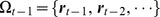, and 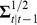 is the symmetric positive definite square root of 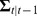. Then a multivariate ARCH process of order 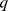 takes the form
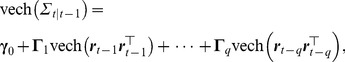
(8)where 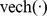 denotes the half-vectorization operator which stacks the columns of a square matrix from the diagonal downwards in a vector, 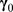 is a 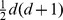-dimensional column vector of constants and 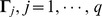 are 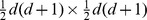 coefficient matrices. It can be seen that even for a bivariate series with a low order, this general model has a fairly large number of parameters. Therefore, more restricted models were proposed. For example, Bollerslev et al. considered diagonal ARCH processes where all the 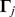 matrices are diagonal [35]. To guarantee the positive definiteness of the conditional covariance matrix 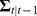, Baba, Engle, Kraft and Kroner investigated the following variant of a multivariate ARCH model, known as the BEKK model [37], [38]

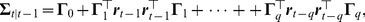
(9)where ^⊤^ is the matrix transpose, 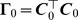 is positive definite, and all 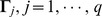 are 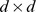 matrices. In contrast to the diagonal model, the BEKK model produces interactions between second order moments and can generate rich volatility dynamics.

We now present a Granger causal model with signal-dependent noise [13]. Consider the following multivariate model with time varying volatility, in particular, signal-dependent noise:
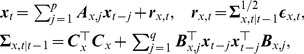
(10)where 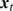 is a 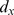-dimensional column random vector, 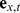 is a 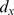-dimensional Gaussian distributed white noise process with zero mean and unit variance, 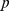 and 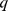 are the model orders, 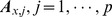, 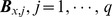 and 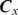 are coefficient matrices. The volatility model is a modification of the BEKK model [38] in which the conditional covariance matrix 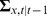 does not regress on the residual process 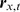 but only depends on the past history of the process 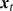 before time 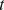. Hence, the covariance (second order statistics) of the noise process is coupled to the mean (first order statistics). This form also guarantees the positive definiteness of 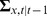. Clearly, when 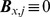 for all 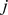, the conditional covariance is time invariant and the model reduces to the AR model. In the light of these points, we term our model (10) the AR-BEKK model.

We now summarize our use of the terms ‘signal’ and ‘noise’ in the reminder of the paper for the model and in the empirical analysis of the fMRI data. We assume that the observed time series 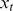 is a realization from the following general process:
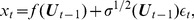
(11)where 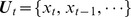, 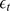 is an i.i.d white noise process, 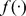 and 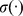 can be any continuous functions, 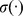 is nonnegative. We define 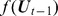 to be the ‘signal’, and 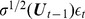 to be the ‘noise’. The variance of the noise is 
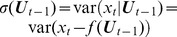
. Given the past history of the time series, the signal is a deterministic process while the noise is what cannot be predicted and produces the variation across realizations. Empirically, since 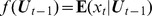, the signal is estimated by projecting 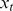 onto the subspace spanned by 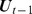. The noise is estimated by the residual of the projection. In the Results section, we investigate different subspaces spanned by 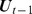 to provide empirical evidence for the signal-dependent noise in fMRI BOLD time series. In the model, we specify particular forms of the functions 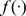 and 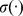. In particular, according to model (10), we assume 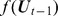 is a linear function of 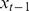, i.e., 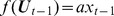, and we assume 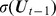 is a quadratic function of 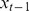, i.e., 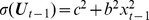. Therefore, in the model, the signal is estimated by 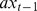 and the variance of the noise is estimated by 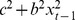. In the Results section, we show the concordance of the definitions of ‘signal’ and ‘noise’ in the model and in the empirical data analysis, that is, in spite of the simplified forms of 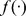 and 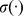, our model captures a large portion of the variance in the empirical signal and noise. We note that classical Granger causality assumes that the variance of the noise as just defined is constant across the time course of the process (e.g., the time course of an fMRI trial), and that Granger causality with signal-dependent noise allows causality to be calculated more powerfully if the variance of the noise within a trial is not constant.

#### Granger causality with signal-dependent noise

To define the causal relationship between 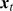 and another 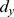-dimensional time series 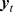, consider the following joint AR-BEKK model:
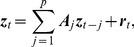
(12)where 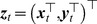, 
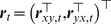
, 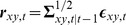, 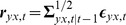, 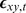 and 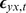 are 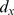-dimensional and 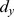-dimensional independent Gaussian distributed white noise processes with zero mean and unit variance respectively, and
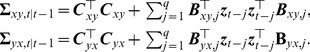
(13)Here, 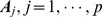, 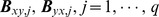 and 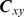, 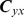 are all coefficient matrices. The causal influence from 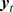 to 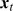 can be defined as [13]

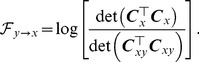
(14)

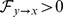
 if 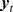 has a causal effect on 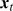, and 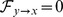 otherwise. It can been seen that 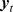 can help improve the prediction of 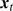 by impacting on either its mean activity through the coefficients in 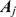, or its variance through the coefficients in 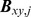. These two cases correspond to causality in the mean and variance respectively. When the noise in 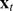 is not signal-dependent, i.e., 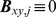 and 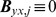 for all 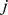, the model reduces to a VAR model and the definition of causality coincides with classical GC in the time domain (See Eq. (4)).

#### Stability conditions

To guarantee the stability of the AR-BEKK model, we provide the stability condition for a simple first-order model, i.e., 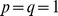 in Eq. (12). This is the model that we usually use for fMRI data analysis considering the poor temporal resolution of BOLD signals, and the relatively fast signal transmission between groups of neurons [2], [4], [39], [40]. In the remainder of this paper, both in simulations and real data analysis, we focus on this first-order model unless otherwise specified. We also assume that 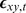 and 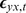 are uncorrelated. The stability of the model involves both the first and second order stability conditions, i.e., the unconditional mean and covariance of 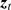 exist and are time invariant. For the first order stability condition, it follows from the theory of the AR model that all the eigenvalues of 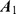 have modulus less than 1. For the second order stability, note that
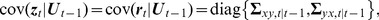
(15)and
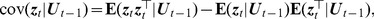
(16)where 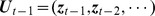. Therefore,
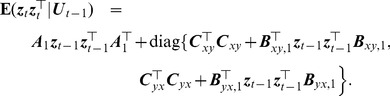
(17)Taking the expectation on both sides yields
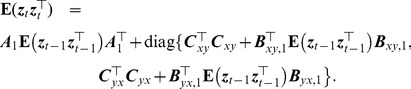
(18)This can be transformed into the following equation using the vectorization operator, which stacks the columns of a square matrix into a column vector:
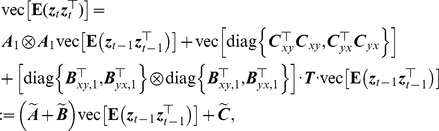
where 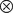 is the Kronecker product,
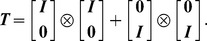
Therefore, it is required that all the eigenvalues of 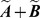 have modulus less than 1.

#### Model estimation

Using Bayes' theorem, the joint density function of 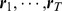 is
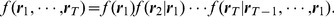
(19)Thus, the conditional distribution of 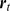 given 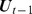 is Gaussian and if the 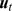 are observed quantities, the log-likelihood function of the AR-BEKK model described by Eq. (12), for a sample 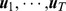 is given by
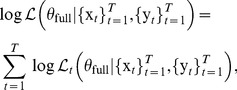
(20)where 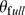 is a vector of all unknown parameters of the model (12) and
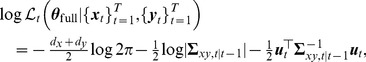
(21)where the required initial values for specifying 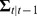 are assumed to be available. The likelihood function may be maximized with respect to the parameters 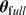 by using numerical methods. Specifically, the initial values of 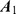 are given by the least square estimates of 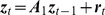, assuming a simple AR model, and 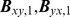 and 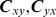 are then initialized to diagonal matrices whose 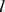-th element on the diagonal is the least square estimate of
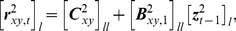
(22)and
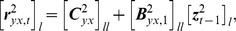
(23)using the residuals 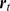 from the AR fitting. The constrained maximum likelihood estimation of the model parameters can be obtained by solving the optimization problem
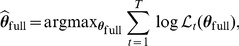
(24)while satisfying the first and second order stability conditions derived above. We use Matlab function fmincon with the interior-point algorithm to tackle this restricted optimization problem. (Note that sometimes fmincon with the interior-point algorithm may fail to converge to a reasonable solution. In this case, we use the active-set algorithm as an alternative.) The parameters of the restricted model (10) can be estimated similarly.

#### Causal inferences

The nonparametric bootstrap method for causal inferences used in [13] is time-consuming. Here we develop an analog of the likelihood ratio test for classical GC (See Eq. (6)) to improve computational efficiency. Similarly, the likelihood ratio test statistic takes the form
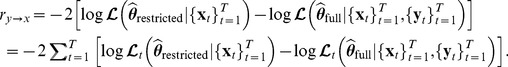
(25)The test statistic approximately follows a chi-squared distribution, and the degrees of freedom are 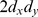. Therefore, a parametric chi-squared test can be carried out to test the significance of the causal influence. This likelihood ratio test also has a connection to the transfer entropy between time series [30]. However, since the residual process in the AR-BEKK model is not a Gaussian white noise, the likelihood ratio test is not equivalent to the measure defined in Eq. (14).

To test the difference between the causalities in the opposite directions between brain areas, note that the difference of the two causality measures is 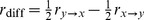, where 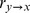 and 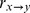 are two chi-squared distributed random variables with the same degrees of freedom. Therefore, the distribution function of 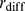 is
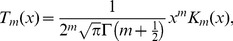
(26)where 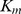 is a modified Bessel function, 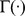 is a Gamma function, and 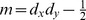 [41]. A table for the two-sided one and five percent quantile of this distribution can be found in [41]. For example, in the investigation of a pair of univariate time series, i.e., 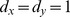, a difference measure of 4.61 implies a 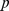-value of 0.01.

### Simulation studies

#### Methodology assessment

We illustrate the Granger causal model with signal-dependent noise and the likelihood ratio test by a simulation study. Consider the following first-order AR-BEKK model for two univariate time series 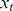 and 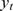:
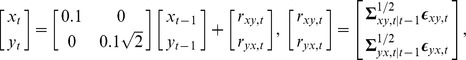


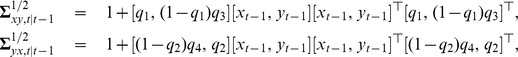
where 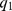 and 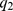 are random numbers uniformly distributed in 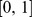, and 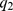 and 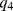 are random numbers with the probability of 0.6 to be 0 and 0.4 to be 1. It is clear that there is a causal influence from 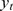 to 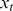 if and only if 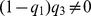, and from 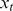 to 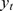 if and only if 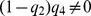.

We generated 100 models with different 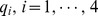, and for each model we generated time series of 1000 points with 2 replicates. We then fitted both the classical Granger causal model and the signal-dependent noise model to the data. Using different 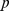-value thresholds, the performance of the two models was compared by the ROC (Receiver Operating Characteristic) curve [42].

#### An illustration of how signal-dependent noise may arise in BOLD activations

In the Results section below, we provide empirical evidence for the presence of signal-dependent noise in fMRI BOLD time series. In order to illustrate how signal-dependent noise may arise in BOLD activations from the underlying neuronal firing, we performed the following simulations. These simulations were on a simple model developed for the purposes of illustration. The concept is to investigate how the close to Poisson firing of neuronal spikes of neurons in the cortex for a given mean firing rate [11], [12] might be reflected in a signal produced by feeding the spiking neuronal activity into a widely used generative biophysical model describing the hemodynamic response [43]. This hemodynamic model links neuronal activity to blood flow and incorporates the well established Balloon model [44], [45]. For the Poisson spiking, the variance of the spike counts in a time window increases linearly with (and is equal to) the mean spike count.

We simulated spike trains of neurons following Poisson processes with mean firing rates of 5 Hz, 40 Hz and 80 Hz respectively for 1 second. The spike trains were then fed into the following biophysical model describing the hemodynamic response induced by the neuronal activity [43]:
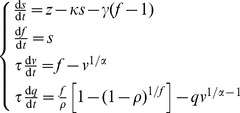
(27)where 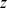 is the input spike trains; 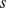 is the vasodilatory signal; 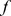 is the cerebral blood inflow (CBF); 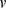 is the cerebral blood volume (CBV); 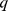 is the deoxyhemoglobin (dHb) content; 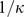 is time constant of signal decay; 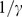 is the time constant of the feedback auto-regulatory mechanism; 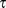 is the mean transit time in the post-capillary venous compartment; 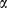 is the Grubb's parameter and 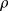 is the resting net oxygen extraction fraction by the capillary bed. Finally, the observed BOLD time series is a nonlinear function of the CBV and dHb content:
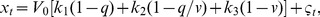
(28)where 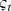 is the observation noise, 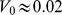 is the resting blood volume fraction, 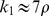, 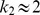, 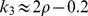 for 1.5-T scanners. All biophysical parameters were set to their typical values (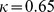; 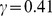; 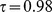; 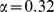; 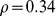) [46]. For each of these firing rates, the simulation was repeated 100 times, producing 100 simulated trials of the fMRI BOLD time series with temporal resolution 1 ms for a total of 25 seconds. We downsampled the time series to a sampling rate of 1 Hz to reflect the temporal resolution of real BOLD signals. We then empirically estimated the signal and noise as defined above. In particular, we investigated different subspaces spanned by 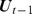, including (1) linear bases with different time lags; (2) second-order polynomial bases with different time lags; and (3) sixth-order Fourier bases with different time lags, to ensure that the observed signal-dependent noise phenomenon does not depend on the selection of the projection space. Any non-random relationship between the empirically estimated signal 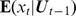 and the empirically estimated variance of the noise 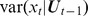 indicates the presence of signal-dependent noise. In particular, we tested if there exists a significant correlation between 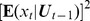 and 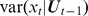.

### fMRI experiment

The fMRI dataset is the same as that obtained and used in previous investigations [17], [47], [48]. We describe key imaging acquisition, preprocessing and psychophysiological interaction (PPI) analyses for completeness. We refer the readers to previous publications for the full details.

#### Participants and ethics statement

Twelve healthy volunteers (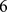 male and 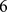 female, age range 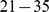) participated in the study. Ethical approval (Central Oxford Research Ethics Committee) and written informed consent from all subjects were obtained before the experiment. The subjects had not eaten for three hours before the investigation.

#### Experimental design

We used the identical taste stimulus, 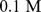 monosodium glutamate (MSG) with 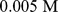 inosine monophosphate (see [49]), referred to throughout this paper for brevity as monosodium glutamate, in two different types of trial. A trial started 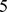 seconds before the taste delivery with the visual attentional instruction either “Remember and Rate Pleasantness” or “Remember and Rate Intensity”, which was shown until the end of the taste period. The 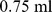 taste stimulus was delivered at 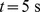. The taste period was from 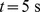 until 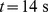, and in this period a red cross was also present indicating that swallowing should not occur. The differences between the activations in this period were a measure of the effects of the top-down selective attention instructions while the taste was being delivered. (We note that in order to utilize top-down attention, one needs to hold the object of attention in mind, in this case pleasantness or intensity. This requires a short-term memory. Short-term memory is thus a sine qua non of selective attention [50], [51], and it is the source of this top-down bias from a short-term memory system in which we are interested in this investigation.) After the end of the taste period, the visual instruction and red cross were turned off, and a green cross was shown cueing the subject to swallow. After 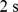 a tasteless rinse was delivered with a red cross, and the rinse period was from 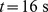 until 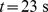, when the green cross appeared to cue a swallow. After this the rating of pleasantness or intensity was made using button-press operated visual analog, rating scales ranging continuously from 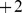 (very pleasant) to 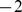 (very unpleasant) for pleasantness, and 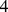 (intense) to 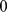 (very weak) for intensity as described previously [52]. These two trial types were interspersed in random permuted sequence with other trials that were part of a different investigation, and each was presented 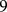 times. As different trial types were being run in the scanner at the same time, and included different stimuli, and no instructions were given about the number of stimuli being used, or that the stimuli were the same on the “Remember and Rate Intensity” and “Remember and Rate Pleasantness” trials, the participants simply had to concentrate on following the instructions about what aspect of the taste stimulus, intensity or pleasantness, had to be rated on that trial. The protocol and design are described in [17], and have been used successfully in previous studies to investigate taste cortical areas [22], [53]–[55].

#### fMRI data acquisition

Images were acquired with a 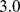-T VARIAN/SIEMENS whole-body scanner at the Centre for Functional Magnetic Resonance Imaging at Oxford (FMRIB), where 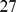 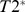 weighted EPI coronal slices with in-plane resolution of 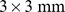 and between plane spacing of 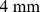 were acquired every 2 seconds (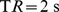). We used the techniques that we have developed over a number of years [49], [53], and as described in detail by [56] we carefully selected the imaging parameters in order to minimize susceptibility and distortion artefact in the orbitofrontal cortex. The relevant factors include imaging in the coronal plane, minimizing voxel size in the plane of the imaging, as high a gradient switching frequency as possible (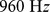), a short echo time of 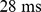, and local shimming for the inferior frontal area. The matrix size was 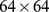 and the field of view was 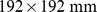. Continuous coverage was obtained from 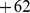 (A/P) to 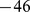 (A/P). A whole brain 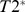 weighted EPI volume of the above dimensions, and an anatomical 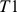 volume with coronal plane slice thickness 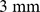 and in-plane resolution of 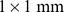 were also acquired.

#### fMRI data preprocessing

The imaging data were analyzed using SPM5 (Statistical Parametric Mapping, Wellcome Trust Centre for Neuroimaging, London. http://www.fil.ion.ucl.ac.uk/spm/). Preprocessing of the data used SPM5 realignment, reslicing with sinc interpolation, normalization to the Montreal Neurological Institute (MNI) coordinate system [57], and spatial smoothing with a 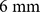 full width at half maximum (FWHM) isotropic Gaussian kernel. Time series non-sphericity at each voxel was estimated and corrected for [58], and a high-pass filter with a cut-off period of 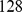 seconds was applied.

#### fMRI data analysis

To investigate task dependent activations of brain areas during the taste period, a Finite Impulse Response (FIR) analysis was performed as implemented in SPM, in order to make no assumption about the time course based on the temporal filtering property of the haemodynamic response function [59], [60]. The *a priori* defined areas of interest (ROI) for which we reported results [17] included brain areas where activations to taste stimuli have been found in previous studies including the medial and lateral orbitofrontal cortex, the pregenual part of the cingulate cortex, and the taste and oral somatosensory parts of the insular cortex [22], [49], [53]–[55], [61], [62]; and areas of the lateral prefrontal cortex where activations related to task set, attentional instructions, and remembering rules that guide task performance have been found, including specifically parts of the middle and inferior frontal gyrus [63]–[70]. A contrast of trials where attention was being paid to taste pleasantness with trials where attention was to intensity revealed significant effects in the orbitofrontal cortex [−6, 14, −20]. The reverse contrast of trials where attention was to intensity vs trials where attention was to pleasantness revealed significant effects in the right anterior insular taste cortex [42, 18, −14] [17].

**Figure 1:**
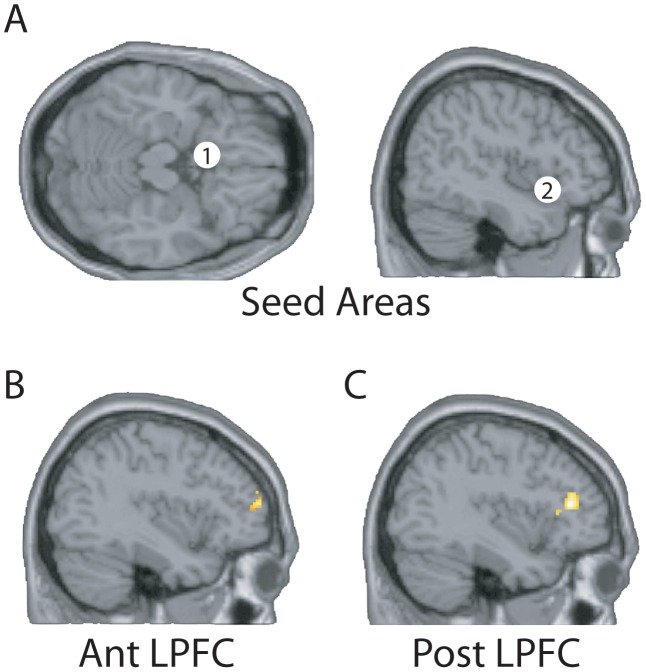
Results of the PPI analysis. A. The seed areas for the PPI analysis in the orbitofrontal cortex (1) [−6, 14, −20], and insular taste cortex (2) [42, 18, −14]. B. The region of the anterior lateral prefrontal cortex (AntLPFC) [−40, 54, 14] identified by PPI analysis as correlated with the orbitofrontal cortex seed area when attention was to pleasantness (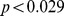). C. The region of the posterior lateral prefrontal cortex [−38, 34, 14] identified by PPI analysis as correlated with the insular taste cortex seed area when attention was to intensity (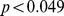). The full details of the PPI analysis are provided in [47].

We then performed PPI analyses [71], [72], using the above two brain areas as seed regions, to investigate task-dependent functional connectivity of these areas with other brain areas, that might provide the source of the top-down modulation [47]. We identified an anterior lateral prefrontal cortex (AntLPFC) region at 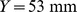 in which the correlation with activity in the orbitofrontal cortex (OFC) seed region was greater when attention was to pleasantness than to intensity [47]. Conversely, in a more posterior region of lateral prefrontal cortex (PostLPFC) at 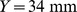 the correlation with activity in the anterior insula (AntINS) seed region was greater when attention was to intensity than to pleasantness [47]. The locations of the seed regions and the identified foci in AntLPFC and PostLPFC are shown in Figure 1.

#### Empirical analysis of BOLD signals

An empirical analysis was performed to provide evidence on whether there is signal-dependent noise in fMRI BOLD time series. Again, we empirically estimated the signal and noise by projecting the current state of the observed fMRI BOLD time series onto a subspace spanned by its past history. We investigated subspaces spanned by different sets of basis functions including (1) linear bases with different time lags; (2) second-order polynomial bases with different time lags; and (3) sixth-order Fourier bases with different time lags, to ensure that the observed signal-dependent noise phenomenon does not depend on the selection of the projection space. Any non-random relationship between the estimated signal 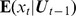 and the estimated variance of the noise 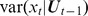 indicates the presence of signal-dependent noise. In particular, we tested if there exists a significant correlation between 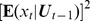 and 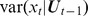. We also investigated the correlation between the empirically estimated signal, 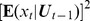, and the model estimate of the signal, 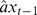; and the correlation between the empirically estimated variance of the noise, 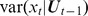, and the variance of the noise estimated by the model, 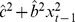, where 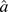, 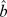 and 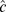 are estimates of the model parameters, to test whether there is good concordance between the model and the empirical data analysis with respect to the signal and noise.

#### Granger causal analysis of fMRI BOLD signals

The PPI analyses described above do not show the directionality of the influences, as they are based on correlations, and for that reason we applied Granger causal analysis to each pair of the four brain areas (OFC, AntINS, AntLPFC and PostLPFC) [48]. We extracted the mean BOLD signals from 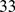 voxels within a sphere of radius 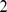 voxels centered at the seed voxels in OFC and AntINS, and the peak voxels identified with the largest PPI effect in AntLPFC and PostLPFC, for Granger causal analysis. For each of the two experimental conditions (attention to intensity vs attention to pleasantness), the time series for a single subject consisted of 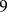 trials, each with 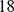 BOLD signal data points (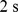 apart), starting on each trial at the onset of the instruction to pay attention to the pleasantness or to the intensity of the taste. Each trial was denoised by wavelet using a Matlab routine, **mswden**, with the Daubechies 2 (db2) wavelet, and threshold options **sqtwolog** (universal threshold at 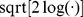) and **sln** (rescaling using a single estimation of level noise, based on first level coefficients). Each trial was also detrended and centered to zero mean before causal analyses. For each experimental condition and each pair of the four brain areas, we pooled data from all subjects (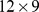 trials) to fit the signal-dependent noise model, i.e., we treated the 108 trials as repeated realizations from a common underlying model. We detected unidirectional causal influences as well as significant difference of the causalities in opposite directions [2] to identify the dominant causal influences in a particular direction [48]. We also applied classical GC to the same data set as a comparison.

## Results

### Simulation results

**Figure 2:**
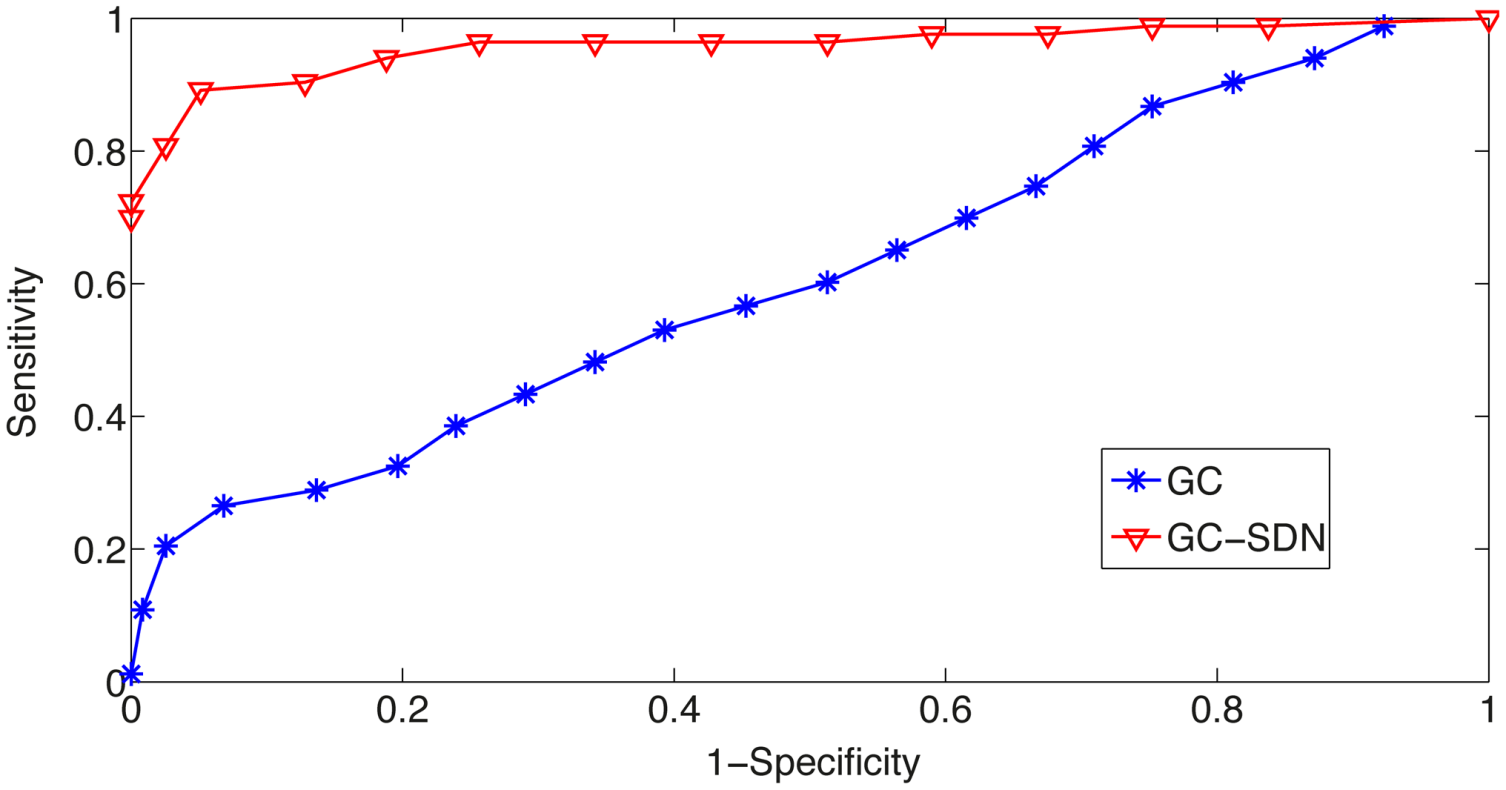
Comparison by simulations of the performance of the classical Granger causal analysis and the Granger causality with signal-dependent noise analysis by ROC (receiver operating characteristic) analysis. The sensitivity of the methods is plotted against 1
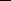
specificity for different 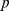-value thresholds. The sensitivity is defined as the proportion of actual causal influences that are correctly identified. The specificity measures the proportion of non-causal influences that are correctly identified. By setting different 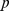-value thresholds for causality, each method gives different sensitivity and specificity. Therefore, the best model is expected to have its performance ROC curve go through the upper left corner, while a random classification algorithm has its performance curve as a diagonal line. The signal-dependent noise model outperforms the classical Granger causal model substantially and consistently.

Figure 2 shows a comparison of performance for the classical Granger causal model and the Granger causal model with signal-dependent noise by ROC (receiver operating characteristic) analysis. Clearly, classical GC cannot capture the causal influences well in the presence of signal-dependent noise, while the signal-dependent noise Granger causal model substantially outperforms the classical GC model, and shows a good sensitivity and specificity.

**Figure 3:**
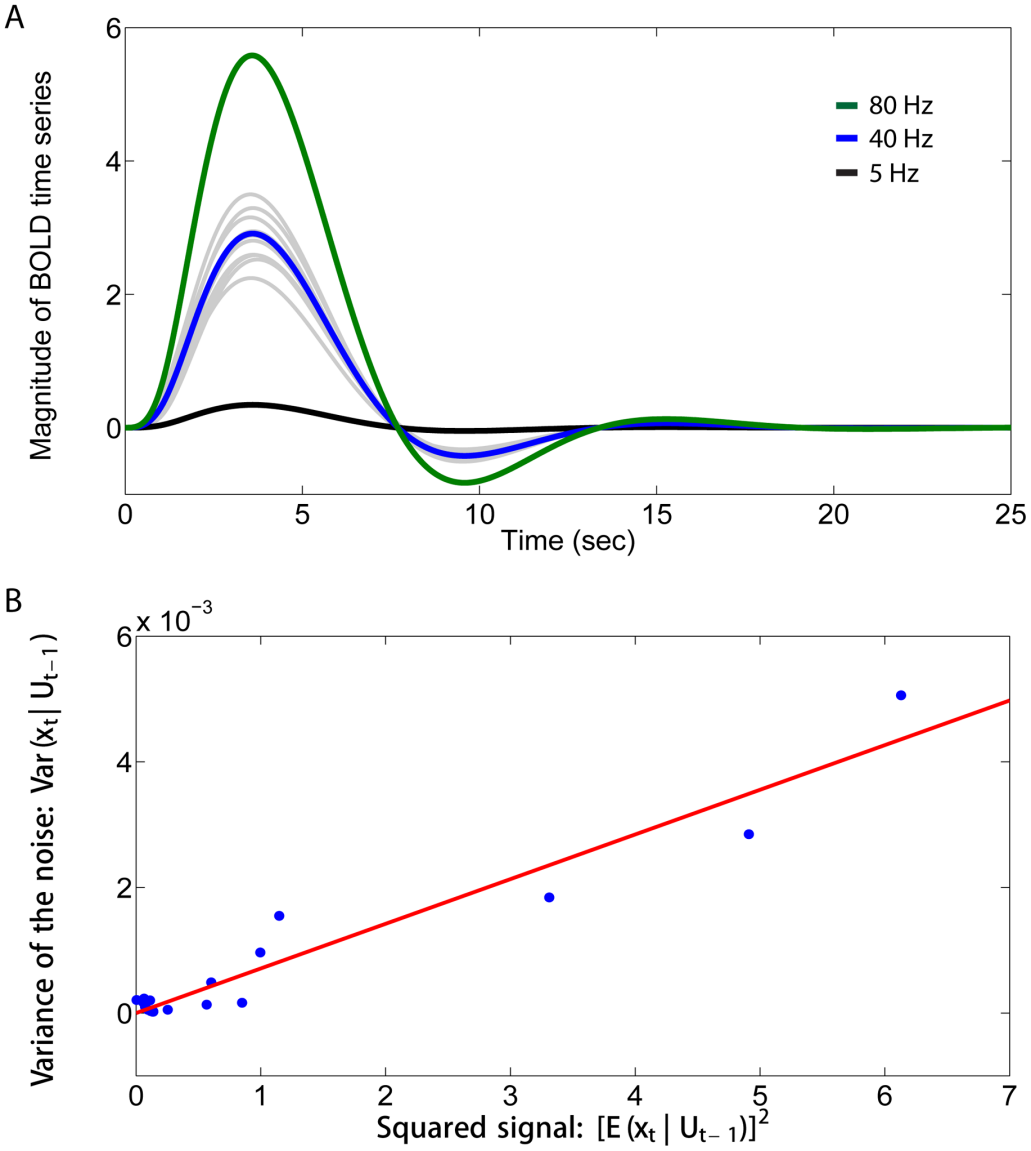
An illustrative model of signal-dependent noise in BOLD signals. A. The mean BOLD signals for the different time points within a trial (calculated across the trials) for three firing rates, with mean rates of 5, 40 and 80 spikes/sec. 10 randomly selected trials of the BOLD signals with the input firing rate of 40 Hz are also shown (gray). B. The empirically estimated variance of the noise in the simulated BOLD time series plotted against the squared empirically estimated signal using the projection space spanned by the second-order polynomial basis, with the input firing rate of 40 Hz. The relation is approximately linear.

Figure 3A shows the mean BOLD signals calculated across the trials for the three firing rates before downsampling. As expected, higher firing rates evoked larger mean modelled haemodynamic responses. However, the variability of the modelled BOLD response was considerable, as illustrated for the mean firing rate of 40 Hz in Figure 3A for 10 randomly selected trials. Figure 3B shows the relation between the empirically estimated variance of the noise and the squared empirically estimated signal at different time points within a trial, using the projection space spanned by the second-order polynomial basis, i.e., 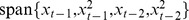. This shows that the variance of the noise at any point in the time course of a trial is approximately linearly related to the square of the signal. We obtained consistent results using different projection spaces. Consistent results with those just described can also be obtained with a simpler model in which the spike trains are convolved with the canonical haemodynamic response function to generate the BOLD signal, as described previously [73], [74]. This simple generative model of BOLD signals thus confirms that Poisson spike trains could produce fMRI BOLD time series in which the variance of the noise across the time course of a trial would be linearly related to the squared signal. We show below that this is also exactly what was found empirically in the fMRI data.

### Empirical evidence for signal-dependent noise in BOLD signals

**Figure 4:**
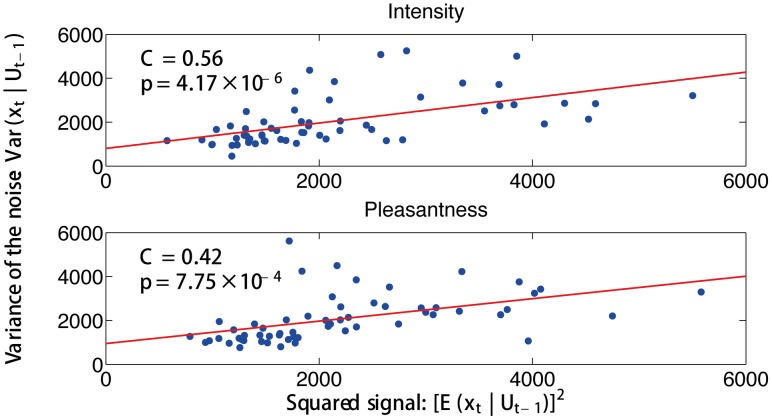
Empirical evidence for signal-dependent noise in BOLD signals. For each of the four brain areas and each subject, the empirically estimated variance of noise in the observed fMRI BOLD time series is plotted against the squared empirically estimated signal for each time point within a trial using the projection space spanned by the second-order polynomial basis. 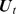 is defined in the text, and reflects the past history of times eries 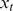. Significant correlations are observed for both experimental conditions (attention to intensity, 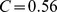, 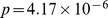, and attention to pleasantness, 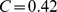, 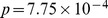).

Figure 4 shows the empirically estimated variance of the noise in the fMRI BOLD time series obtained in this investigation as a function of the squared empirically estimated signal at each time point within a trial, using the projection space spanned by the second-order polynomial basis, i.e., 
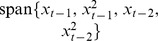
. Significant correlations are observed for both experimental conditions by pooling data from the four brain regions (attention to intensity, 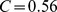, 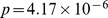, attention to pleasant, 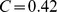, 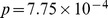), which clearly indicates the presence of signal-dependent noise in the fMRI BOLD time series. In particular, the results shown in Figure 4 show that the variance of the noise in BOLD time series is approximately linearly related to the squared signal. A similar effect was also found for each brain region when analyzed separately. The results were consistent using different projection spaces. In particular, we observed significant correlations when the project space was spanned by (1) linear bases up to 9 time lags; (2) second-order polynomial bases up to 6 time lags; and (3) sixth-order Fourier bases up to 2 time lags. These results provide strong evidence for the presence of signal-dependent noise in fMRI BOLD time series. Moreover, when fitting our signal-dependent noise model to the real data, we observed excellent concordance and significant correlation between the empirically estimated signal, 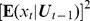, and the model estimate of the signal, 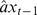 (attention to intensity, 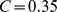, 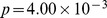, attention to pleasantness, 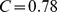, 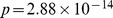), and between the empirically estimated variance of the noise, 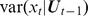, and the variance of the noise estimated by the model, 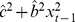 (attention to intensity, 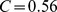, 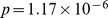, attention to pleasantness, 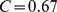, 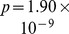). The results were also consistent using different projection spaces. This indicates that the AR-BEKK model is a good description of the data and captures a large portion of the variance in the empirical signal and noise.

### fMRI data investigation

Table 1 shows the causal influences between the four brain areas (OFC, AntINS, AntLPFC, PostLPFC) detected by the Granger causality with signal-dependent noise analysis. First, we consider attention to intensity. There are significant (top-down) causal influences from both the AntLPFC and PostLPFC to the insular taste cortex (AntINS). Second, we consider attention to pleasantness. There are significant (top-down) causal influences from both the AntLPFC and PostLPFC to the OFC, and a significant effect from the OFC to the antLPFC. There is also a (top-down) effect of the PostLPFC on the taste insula (AntINS). Very interestingly too, during attention to pleasantness, there is increased effective connectivity from the insular taste cortex to the OFC, suggesting that information is routed especially to the OFC during attention to pleasantness.

**Table 1:** Causality results by Granger causality with signal-dependent noise analysis.

Intensity				
	OFC	AntINS	AntLPFC	PostLPFC
**OFC**	–	4.51 (0.10)	0.96 (0.62)	0.68 (0.71)
**AntINS**	2.15 (0.34)	–	**28.57 (** 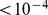 **)**	13.59 ( 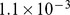 )
**AntLPFC**	0.89 (0.64)	**28.44 (** 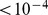 **)**	–	5.17 (0.08)
**PostLPFC**	0.62 (0.73)	**29.62 (** 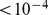 **)**	4.22 (0.12)	–

The causal influence is from row to column. The causality is given for each direction, and the corresponding 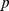-value is presented in brackets. If the uncorrected 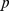-value is less than 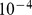 (surviving Bonferroni correction), the causal influence is identified as significant and indicated in bold in the table. (*: The active-set algorithm was used.)

For comparison, Table 2 shows the causal influences between the four brain areas detected by the classical Granger causal model. Only one effective connectivity influence (PostLPFC to AntLPFC, when paying attention to intensity) was identified as significant. The greater power of the signal-dependent noise model can be clearly observed.

**Table 2:** Causality results by classical Granger causal analysis.

Intensity				
	OFC	AntINS	AntLPFC	PostLPFC
**OFC**	–	0.0039 (0.0061)	0.0006 (0.30)	0.0001 (0.62)
**AntINS**	0.0015 (0.09)	–	0.0002 (0.56)	0.0003 (0.42)
**AntLPFC**	0.0000 (0.99)	0.0056 (0.0009)	–	0.0043 (0.0041)
**PostLPFC**	0.0003 (0.43)	**0.0122 (** 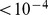 **)**	0.0013 (0.12)	–

Pleasantness				
	OFC	AntINS	AntLPFC	PostLPFC
**OFC**	–	0.0000 (0.99)	0.0001 (0.71)	0.0000 (0.98)
**AntINS**	0.0001 (0.74)	–	0.0023 (0.03)	0.0018 (0.06)
**AntLPFC**	0.0000 (0.99)	0.0002 (0.51)	–	0.0059 (0.0007)
**PostLPFC**	0.0008 (0.22)	0.0034 (0.0099)	0.0002 (0.59)	–

The causal influence is from row to column. The causality is given for each direction and the corresponding 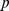-value is presented in brackets. If the uncorrected 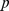-value is less than 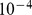 (surviving Bonferroni correction), the causal influence is identified as significant and indicated in bold in the table.

Table 3 shows the difference of the causalities in opposite directions by the Granger causality with signal-dependent noise analysis. In the pleasantness condition, consistent with the hypothesis that the lateral prefrontal cortex is the source of the top-down modulation of activations in the OFC, there are significantly stronger effects from both the AntLPFC and the PostLPFC to the OFC than vice versa. It is also of interest that in the pleasantness condition, a significantly stronger forward influence was detected from the antINS to the OFC. Only one significant difference was detected for the intensity condition, that is the effect from the PostLPFC to AntINS is greater than in the reverse direction. This is consistent with the hypothesis that the major top-down effect on the taste insula during attention to intensity is from the PostLPFC. The bi-directional interaction in the pleasantness condition between the AntLPFC and OFC (Table 1) may be interpreted in the context that there is a significant difference of the causality with AntLPFC to OFC greater than OFC to AntLPFC, thus indicating a stronger influence of AntLPFC on OFC than vice versa (Table 3).

**Table 3:** Difference of the causalities in opposite directions by the Granger causality with signal-dependent noise analysis.

Direction	Intensity	Pleasantness
PostLPFC→AntLPFC – AntLPFC→PostLPFC	−0.48	−0.614*
PostLPFC→AntINS – AntINS→PostLPFC	**8.02**	2.62
PostLPFC→OFC – OFC→PostLPFC	−0.03	**41.03**
AntLPFC→AntINS – AntINS→AntLPFC	−0.06	2.32
AntLPFC→OFC – OFC→AntLPFC	−0.03	**25.07**
AntINS→OFC – OFC→AntINS	−1.18	**38.93**

Differences with a 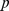-value smaller than 0.01 (a difference measure greater than 4.61) are indicated in bold. (*: We used the active-set algorithm for this particular link.)

**Figure 5:**
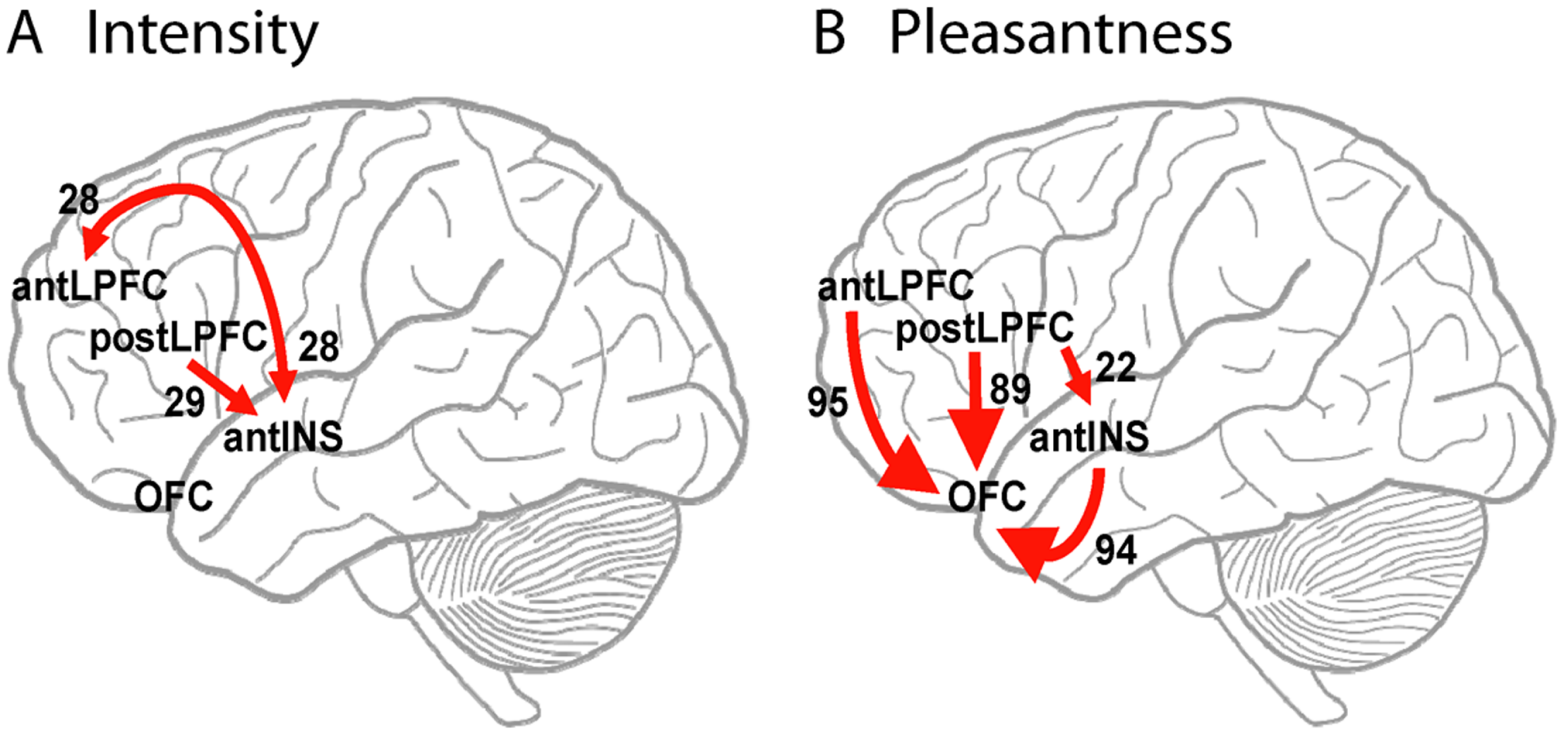
Neural circuits revealed by Granger causality with signal-dependent noise. A. Attention to taste intensity. B. Attention to taste pleasantness. Larger arrows represent a stronger influence. The values of significant likelihood ratio test statistics are indicated.

These analyses provide evidence for the effective connectivities in the attention to intensity and pleasantness conditions that are summarized in Figure 5.

## Discussion

In this paper, we for the first time provide empirical evidence for signal-dependent noise in fMRI BOLD signals in several cortical areas, such as the insular, orbitofrontal, and lateral prefrontal cortical areas. We then developed a Granger causal model with signal-dependent noise that can appropriately model BOLD signals and detect causal influences in both mean and variance. By simulation studies, we showed that our Granger causality with signal-dependent noise analysis substantially outperforms classical Granger causal analysis, when signal-dependent noise is present in the time series. We applied our Granger causal model with signal-dependent noise to the data from an fMRI study to investigate the source of the top-down attentional influences on taste processing when attention was to the intensity vs the pleasantness of the taste. We found a top-down effect from the PostLPFC to the insular taste cortex during attention to intensity but not to pleasantness; and a top-down effect from the AntLPFC and PostLPFC to the OFC during attention to pleasantness but not to intensity. In addition, there was stronger forward effective connectivity from the insular taste cortex to the OFC during attention to pleasantness than during attention to intensity.

### Assessment of the measurement of Granger causality taking into account signal-dependent noise

Conditionally heteroskedastic data often show volatility clustering and outliers. In particular, the unconditional distribution of the data is leptokurtic, which means that it has more mass around zero and in the tails than the normal distribution and, hence, it can produce occasional outliers [27]. Therefore, models with time varying volatility can better capture the nature of the data, and it is expected that more reliable causal inferences can be made. Comparing to the earlier approaches of causal inferences in data with time varying volatility [75]–[78], including [13], the model presented in this paper that takes into account signal-dependent noise provides an accurate, efficient and unified method to detect causality in both the mean and variance. The model has a corresponding frequency domain representation [13], which may further shed light on frequency-specific interactions. The model described here applies when the variance of the noise is proportional to the square of the signal, which is what we observed from real fMRI BOLD time series, but could in principle be extended to deal with other cases. There are alternative measures of Granger-type causality such as partial directed coherence (PDC) [79], relative power contribution (RPC) [80] and directed transfer function (DTF) [81] that do not explicitly use the noise covariance function to define causality but are based on the transfer function or model coefficients. However, because they are typically formulated under the simple AR model, all these methods are unable to capture causal influences in the second order statistics such as signal-dependent noise.

It is not easy to tease apart ‘signal’ and ‘noise’ from an observed time series. In this paper, we define ‘signal’ as the part of the observations that can be well predicted from the past history of the time series, and ‘noise’ as what is completely unpredictable and produces the variation across realizations. We therefore empirically estimate the signal by projecting the current state of the time series onto the subspace spanned by its past history. In practice, if the projection space is not constructed appropriately, part of the signal that does not lie in the projection space may migrate to the residual process and produce artificial signal-dependent noise phenomena. In this case, expanding the projection space, i.e., using a more complex model to describe the mean activity of the time series, may mitigate the issues of signal-dependent noise. However, if the variance of the noise is indeed dependent on the signal, simply increasing the complexity of the model in the mean structure will not remove this dependence. In the present paper, we investigated a number of projection spaces, spanned by linear or nonlinear basis functions with different time lags, and always observed signal-dependent noise. Therefore, there is strong evidence for the presence of signal-dependent noise in fMRI BOLD time series. In particular, the variance of the noise is approximately linearly related to the square of the signal. When constructing our signal-dependent noise model, we made use of this relationship and specified a linear function to describe the mean activity of the observations, and a quadratic function for the variance of the noise. Although the model appears to be simple, we have shown that it captures a large portion of the variance in the signal and noise in the empirical BOLD time series. Future studies will be of interest to provide more evidence on signal-dependent noise in different brain areas and different data sets, to further examine the relationship between the variance of the noise and the signal, and to develop more complex models, e.g., using nonlinear functions or kernels, accordingly.

Although we only applied our model to fMRI time series, it is clear that the model can be applied to very many types of data that might exhibit signal-dependent noise, including neurophysiological data such as single or multi-neuron recordings, magnetoencephalography, local field potentials, and beyond neuroscience also to any possibly causal system where there are time series of data from two or many sources. Indeed, the significance of detecting causality from data with time varying volatility might be partly demonstrated in the 2003 Nobel Prize in Economics shared by Granger, who set up the foundation of Granger causal analysis [6], and Engle, who invented the first changing volatility model [31].

Although our initial implementation of the signal-dependent noise model appears to be successful, due to the highly nonlinear form of the log-likelihood function and optimization problem, fast and robust optimization algorithms deserve future investigation. Also, although a low-order low-dimensional AR-BEKK model is a relatively parsimonious representation of the conditional covariance structure of a process, the number of parameters still grows quickly with the dimension of the underlying system. This impedes the application of the model to a modest number of time series. Future studies are needed to find more restricted models that ensure uniqueness of the parameterization, guarantee the positive definiteness of the conditional covariance, while at the same time still produce rich dynamics.

In spite of the wide and successful applications in neurophysiological data, there is still an ongoing debate on applying GC to fMRI data [10], [82]–[87]. Inferring causality from fMRI time series — an indirect measure of neuronal activities – imposes many more challenges than direct electrophysiological recordings. Granger causal models use the observed fMRI data as a surrogate for the underlying neuronal activity, which is a potential flaw of the method and the main controversy against the application of GC to fMRI data, since the BOLD signal is a blurred and delayed representation of the original neuronal signal, and it is now widely recognized that there is intra- and inter-subject variability of haemodynamic responses [88]–[91]. However, there have been a series of numerical and theoretical works showing that GC is quite robust to the difference in haemodynamic delays [92]–[94]. Moreover, as in [4], we calculated the cross-correlation function for each pair of time series used in our Granger causal analysis, and most of the cross-correlation peaks appeared at zero lag, indicating that differences in the regional haemodynamic responses may not be a significant factor in this study. We therefore feel that the application of Granger type causal inferences in the analysis of this particular fMRI data set is justified. However, given the complexity of the brain, much work remains to do to provide reliable and accurate causal analyses for neuroscience.

### Neural interpretation

The interpretation of the effective connectivity revealed with our signal-dependent noise model is that during attention to pleasantness, the AntLPFC and PostLPFC regions identified by PPI analysis exert a top-down control of the responsiveness of the OFC to its taste-related inputs, and indeed to how strongly information is routed to the OFC from its preceding area, the AntINS taste cortex. In contrast, during attention to intensity, the PostLPFC identified by PPI analysis exerts a top-down control of the responsiveness of the insular taste cortex to its taste-related inputs. This interpretation is strengthened by the findings with our componential Granger causal analysis [48], which provides evidence that the top-down effects depend on the level of activity in the areas on which there is a top-down effect.

**Figure 6:**
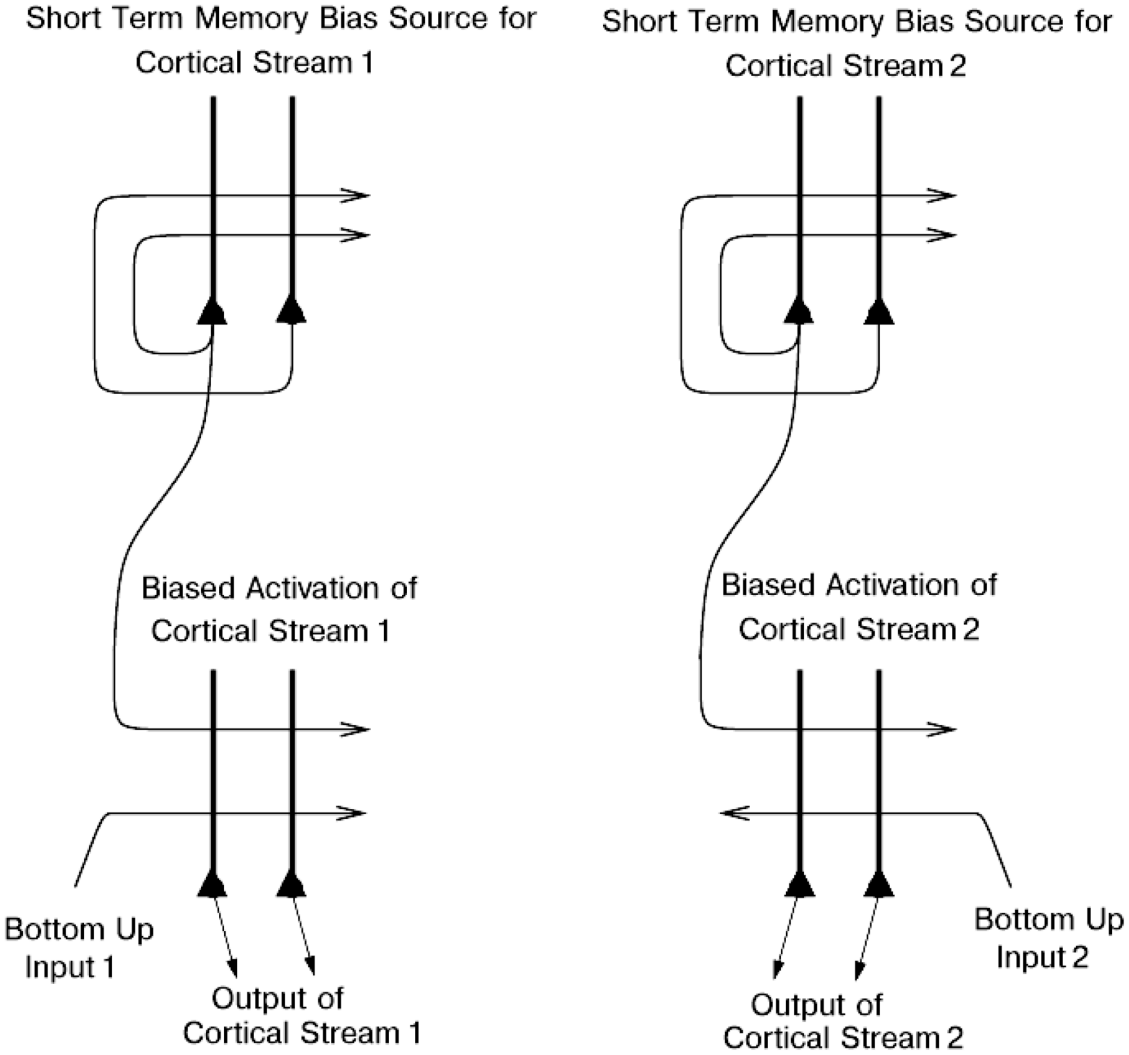
A Biased activation theory of selective attention. The short-term memory systems that provide the source of the top-down activations may be separate (as shown), or could be a single network with different attractor states for the different selective attention conditions. The top-down short-term memory systems hold what is being paid attention to active by continuing firing in an attractor state, and bias separately either cortical processing system 1, or cortical processing system 2. This weak top-down bias interacts with the bottom up input to the cortical stream and produces an increase of activity that can be supralinear [98]. Thus the selective activation of separate cortical processing streams can occur. In the example, stream 1 might process the affective value of a stimulus with the areas involved including the anterior lateral prefrontal cortex with a top-down influence on the orbitofrontal cortex, and stream 2 might process the intensity and physical properties of the stimulus with the areas involved including the posterior lateral prefrontal cortex with a top-down influence on the insular taste cortex. The outputs of these separate processing streams then must enter a competition system, which could be for example a cortical attractor decision-making network that makes choices between the two streams, with the choice biased by the activations in the separate streams [19].

The way that we think of top-down biased competition as operating normally in, for example, visual selective attention [95] is that within an area, e.g. a cortical region, some neurons receive a weak top-down input that increases their response to the bottom-up stimuli [95], potentially supra-linearly if the bottom-up stimuli are weak [50], [51], [63]. The enhanced firing of the biased neurons then, via the local inhibitory neurons, inhibits the other neurons in the local area from responding to the bottom-up stimuli. This is a local mechanism, in that the inhibition in the neocortex is primarily local, being implemented by cortical inhibitory neurons that typically have inputs and outputs over no more than a few mm [50], [51], [96]. This model of biased competition is illustrated in [47]. That locally implemented biased competition situation may not apply in the present case, where we have facilitation of processing in a whole cortical area (e.g. orbitofrontal cortex) or even cortical processing stream (e.g. the linked orbitofrontal and pregenual cingulate cortex [47]) in which the activity of taste neurons may reflect pleasantness and not intensity. So the attentional effect might more accurately be described in the present case as biased activation, without local competition being part of the effect. This biased activation theory and model of attention, illustrated in Figure 6, is a rather different way to implement attention in the brain than biased competition, and each mechanism may apply in different cases, or both mechanisms in some cases [19], [47], [97].
